# Multi-PW laser–driven proton acceleration using a plasma-lens target

**DOI:** 10.1038/s41598-025-29793-7

**Published:** 2025-12-06

**Authors:** Vojtěch Horný, Domenico Doria

**Affiliations:** 1 Extreme Light Infrastructure - Nuclear Physics, IFIN-HH, 30 Reactorului Street, 077125 Magurele, Romania; 2 Faculty of Nuclear Sciences and Physical Engineering, Czech Technical University in Prague, Břehová 7, 115 19 Prague, Czech Republic

**Keywords:** Laser plasma, Ion acceleration, Double layer target, Radiation pressure acceleration, Laser-produced plasmas, Plasma-based accelerators

## Abstract

We investigate laser-driven proton acceleration using state-of-the-art multi-petawatt laser technology and a double-layer target design. The front layer is composed of homogenised near-critical density carbon, which enhances the laser pulse through relativistic self-focusing, effectively acting as a lensing medium. This layer is paired with a solid plastic rear layer that serves as the primary acceleration medium. The thicknesses of both layers and the density of the front layer are optimised to maximise acceleration efficiency of the solid layer protons. These protons are accelerated up to 550 MeV through a synergistic interplay of acceleration mechanisms, with hole boring and light sail radiation pressure acceleration playing dominant roles. These mechanisms are further enhanced by target normal sheath acceleration, which benefits from increased laser-to-electron coupling, especially in the front near critical density part. Additionally, proton acceleration is accompanied by the generation of $\gamma$-ray radiation via nonlinear inverse Compton scattering. Our investigation employs fully resolved 3D particle-in-cell simulations, providing comprehensive insights into the underlying dynamics. Detailed technical aspects of the simulation setup are discussed.

## Introduction

The progress made in compact laser-based ion accelerators over the two past decades^1,2^ has opened up promising perspectives across various research areas, including radiography of fast-evolving dense plasmas and electromagnetic fields^3–6^, investigations into warm dense matter^7–9^ and medical applications^10,11^. Currently, high-power laser systems can deliver pulses of tens of fs duration as powerful as several PW, enabling reaching a peak intensity of the order of $\gtrsim 10^{22}$ W cm^-2^ and even higher. Nevertheless, despite such a tremendous achievement in pulsed laser intensity, the actual peak proton energy attained remains behind expectation. The current record in the proton energy of 150 MeV has been achieved with a 1 PW laser pulse at the DRACO laser system^12^. Plastic targets ($\approx$250 nm thick) were driven to the onset of relativistically induced transparency by the main pulse, following pre-expansion due to irradiation by laser pre-pulses and the pulse rising edge. As a result, a cascade of the acceleration mechanism such as hole-boring Radiation Pressure Acceleration (RPA)^13–15^, relativistic transparency front RPA^16^ and collisionless shock acceleration^17^ at the laser front side, and target normal sheath acceleration (TNSA) at the laser rear side has been identified to contribute to the total acceleration. Up to PW level^12^, the scaling suggests that the proton cut-off energy $\varepsilon$ grows linearly with the laser pulse energy, i.e. $\varepsilon \propto E_l$, enhancing the previous scaling $\varepsilon \propto E_l^{0.7}$ reported in Ref.^18^ for the regime of the single layer target on the onset of the relativistic transparency. Therein, a simple relation

1 $$\begin{aligned} L_{opt}=0.5\lambda a_0 \frac{n_c}{n_e} \end{aligned}$$

linking the optimum target thickness $L_{opt}$ and plasma electron density $n_e$ normalised by a critical density $n_c$ and the laser strength parameter $a_0$ has been found for the case of an unperturbed free-standing foil target irradiated by the ideal Gaussian beam.

**Fig. 1:**
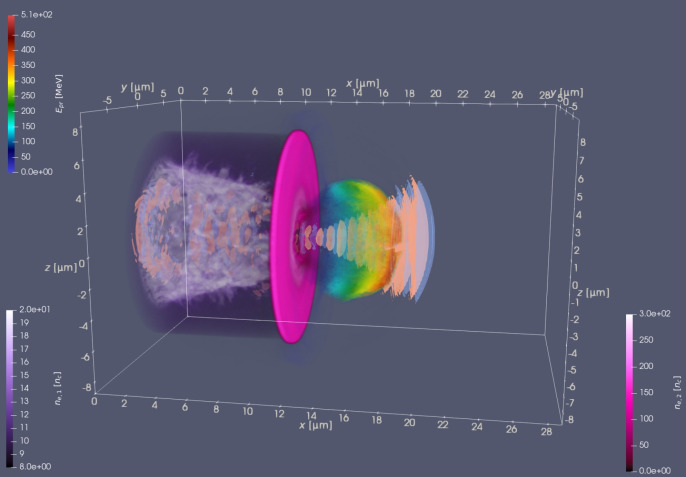
Scheme setup as the visualisation of the 3D PIC simulation at the time $t=130$ fs. The shades of purple and pink correspond to the electron densities in units of critical density for the NCD and solid layers, respectively. The rainbow colourmap depicts the average energy of the accelerated protons. The light red and light blue isosurfaces emphasise the electric field $E_y = \pm 120$ TV/m. Full movie available in the Supplementary Material (Movie 1).

Nevertheless, it is questionable whether it is possible to rely on such a scaling also when considering multi-PW systems such as ELI-NP^19^, Apollon^20^, or CORELS^21^. There, the intensity of various prepulses and a rising edge of the pulse within up to a few picoseconds before the main pulse arrival should increase correspondingly to the intensity of the main pulse, causing premature ionisation and subsequent expansion of the target. As a result, the main pulse then likely interacts with a target which is effectively thicker and less dense, compromising the acceleration efficiency. Potential measures which might mitigate this issue are in use; the most considered is the usage of single or double plasma mirrors^22,23^ or various plasma shutters^24^, effectively filtering the prepulse and even shaping or manipulating the main pulse to improve the acceleration process further.

For an illustration, during the commissioning campaign at the 10 PW wing of the ELI-NP, the proton cut-off energy of 150 MeV has been achieved with a few hundreds nm thick targets^19^. There, the protons have perhaps been accelerated via the standard TNSA mechanism, exhibiting the typical scaling $\varepsilon \propto E_l^{0.5}$ predicted by the TNSA models^25,26^. The literature has extensively confirmed such a scaling under many different parametric conditions. It can be further adjusted empirically^27^, considering the effects of the pulse duration, spot size, and target thickness. In general, the TNSA with 10 PW pulses generates a sheath field exceeding several TV/m, accelerating the protons to energies of the order of a few hundred MeV.

In this work, we investigate an advanced proton acceleration scheme in the multi-PW regime. A 3D PIC representation is illustrated in Fig. 1. Specifically, we consider double-layer targets comprising a near-critical-density (NCD) layer on the laser-irradiated side, attached to a solid-density layer that contains the ions to be accelerated. The front NCD layer serves threefold purposes. First, it acts as an effective shield, protecting the rear solid layer from degradation due to laser prepulses. Second, it functions as a lens, intensifying the laser pulse. If properly designed, this NCD layer enables the laser pulse to self-focus, enhancing its intensity several times at the cost of some energy loss^28,29^. Lastly, the front layer serves as a reservoir of the hot electrons, enhancing the acceleration, especially in its latter stage.

To disentangle the effects of laser pulse intensification from those of proton acceleration, and to remain consistent with previous theoretical and experimental studies^29–33^, we restricted protons to the solid layer and modelled the NCD layer as a homogeneous carbon plasma. This choice provides a controlled framework to isolate the role of the NCD plasma lens. We note, however, that protons in the NCD layer can also be efficiently accelerated, for example via channel acceleration^34^ or, to some extent, magnetic vortex acceleration^35^. Optimising the NCD layer for such mechanisms requires different design parameters than those considered here and will be the subject of follow-up studies.

The idea to use various types of double-layer target configurations has been explored earlier with several motivations. For instance, Hata *et al.*^36^ suggested attaching to a thin high-Z foil such a proton-containing layer which is thicker than the contaminant, to suppress its expansion before the main pulse arrival due to the influence of the rising edge preceding the main intense pulse. To a certain extent, this work further develops the findings from the Refs.^32,37^ aimed at the TNSA with double-layer targets where the front NCD serves as the plasma lens and/or the source of hot electrons.

In this work, we investigate the multi-petawatt regime, exploiting advanced acceleration mechanisms. Simulations are performed using a finely resolved computational grid, which allows the full solid-layer density to be modeled without reduction, thereby ensuring the high fidelity and accuracy of the results.

We describe a configuration where the laser pulse intensity is enhanced by a factor of $6-10\times$ in the NCD layer, reaching peak values up to $4.5\times 10^{23}$ W cm^-2^. This intense pulse accelerates protons from the solid-density layer to energies of up to 550 MeV. The resulting proton energy spectrum is broadband and decreases slower than exponential with high energy. Remarkably, within a 10% range around an energy of 300 MeV, approximately $1.3\times 10^{10}$ protons are accelerated, potentially representing a significant step towards parameters required for medical applications^38^.

## Results

This results section is structured in the following way. First, the laser propagation in the near-critical density layer is scrutinesed, searching for an optimum design for the laser pulse intensification. The prediction on the proton acceleration with such target is introduced subsequently, followed by the analysis of the acceleration processes. All the results have been obtained by three-dimensional particle-in-cell simulation with the code smilei^39^. The laser parameters are chosen to imitate the state-of-the-art multi-PW ultrashort laser pulses. Such a pulse carries the energy of $\mathcal {E}_{l,0}=122$ J with the duration at full width-half maximum and minimum waist of $\tau =25$ fs and $w_0 = 2.55$ $\upmu$m, respectively. It correspondes to to a peak power of 4.6 PW and the intensity of $4.5\times 10^{22}$ W cm^-2^. Detailed information on the physical and numerical parameters is given in the Methods section.

### 3D simulations of laser intensification in the near-critical plasma

**Fig. 2:**
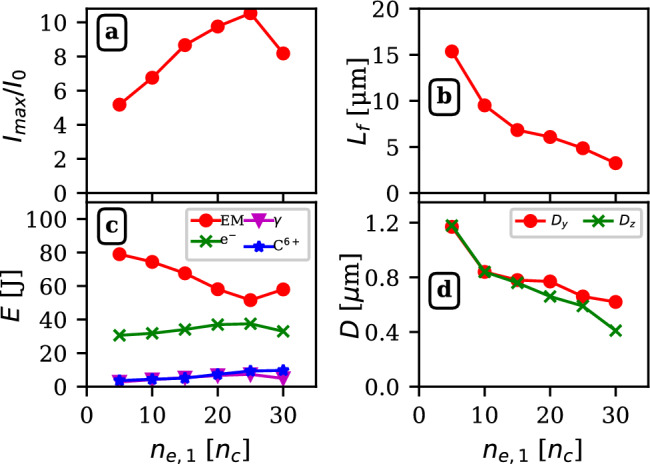
Laser pulse intensification in the near-critical density plasma. The dependence on the electron density of the a) maximum intensification factor, and b) depth in NCD, c) energies stored in the electromagnetic fields (EM, circles), electrons ($\hbox {e}^-$, crosses), photons ($\gamma$, triangles), and carbon ions ($\hbox {C}^{6+}$, stars), and d) FWHM spot sizes along the $y-$ and $z-$-axis at the time of the maximum intensification is reported.

In this Section, we investigate the laser pulse intensification in carbon-based NCD layer. The results are presented in Fig. 2, where panels (a-d) show the dependence of the intensification factor, the focusing length, the energy stored in electromagnetic fields (EM) and different particle species, and FWHM spot sizes in transverse directions on the plasma electron density in the range of [5, 30] $n_{c}$. All data refer to the time of occurrence of a peak intensification which might happen earlier than the peak focusing, especially in the higher densities when the losses from absorption and reflection impair the strength of the peak intensity in the focal point.

Panel a) shows a linear dependence of the intensification factor with the plasma density up to a maximum at $n_{e,1}=25n_{cr}$ where an intensification factor of $I_{max}/I_0=10.5$ is reached. Panel b) shows a monotonic decrease of the distance where a maximum intensification occurs, with the plasma density in the range from 15.3 $\upmu$m to 3.2 $\upmu$m. The energy balance, shown in panel c) highlights several observations. First, it shows that the energy absorbed by electrons does not vary significantly with the plasma density and remains in the range of [30, 38] J. The energy transferred to carbon ions or $\gamma -$photons always remains below 10 J. Also, while, the carbon ions total energy slowly linearly increases with the plasma density, electron and gamma exhibit a trend similar to the intensification factor also peaking at $n_{e,1}=25 n_{c}$, resulting in the generation of the fastest electrons, maximizing photon emission thereby. Panel d) shows the FWHM dimensions of the spot in the *y* and *z* directions. These decrease with the density from 1.2 $\upmu$m to sub-wavelength values.

### 3D PIC simulation of the proton acceleration

Here, the proton acceleration employing the double layer target configuration is investigated. The density and the thickeness of the both plasma layers have been selected based on the investigation carried out in the previous section, and on the results from preliminary 3D simulations run with a more coarse numerical resolution (shown in Supplementary material). In particular, the length and the electron density of the front foam layer are $L_1=9.6$ $\upmu$m and $n_{e,1}=10n_{c}$, respectively. Such a choice represents a reasonable compromise between the intensification of the laser pulse (by a factor of $I/I_0=6.75$) and energy losses due to the dissipation of the laser energy in the front plasma layer (cf. Fig. 2). The thickness of the rear layer is set to 450 nm; that is smaller than Brantov’s optimum condition for a single foil target thickness (1) which is 820 nm. This choice results in a greater proton acceleration, as supported by preliminary simulations with a more coarse resolution presented in Supplementary Material). There, the physical reason for such a better acceleration from a thinner solid layer in a double layer configuration than from a freely standing single foil case is discussed. Briefly, it is an enhanced TNSA component of the acceleration process sustained by a continuous supply of hot electrons from a NCD layer. Such a phase occurs after the laser pulse penetrates the light sail^40^ accelerated plasma front.

The 3D visualisation of the simulation at the time $t=130$ fs is shown in Fig. 1. The shades of purple and pink display the electron density in the units of critical density for the NCD and solid parts of the target, respectively; the light blue and light red isosurfaces of the $E_y$ field represent the laser pulse and the rainbow colormap shows the local average energy of protons. In the NCD layer, it is evident the trace left by the self-focusing process in the form of a shock wave propagating along a radial direction, with a conical shape with the narrowest part close to the solid layer. A fraction of the laser pulse also propagates through the initially opaque solid layer, contributing to the acceleration in th hybrid TNSA/RPA regime^41^.

**Fig. 3:**
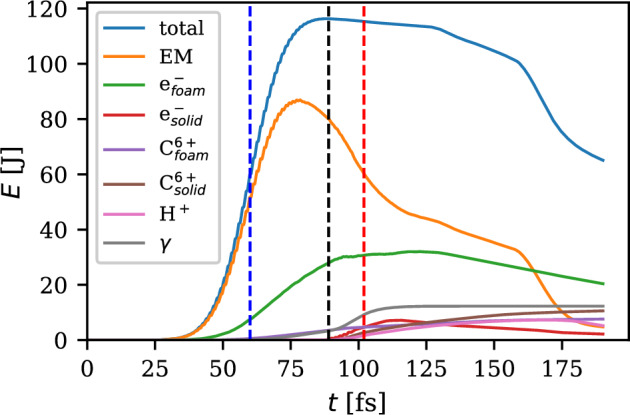
Energy balance during the entire simulation. Blue - total energy in the box, orange - energy in electromagnetic fields, other colours - kinetic energies of different particle species, and the energy of the emitted $\gamma$- photons.

The energy balance during the entire simulation is shown in Fig. 3. The vertical dashed lines approximately mark the time instances when the middle of the laser pulse enters the simulation box (blue), hits the solid foil (black) and when the onset of relativistic transparency is achieved (red). The blue solid curve marks the total energy in the simulation box. Despite the energy of 122 J has been delivered to the box by the laser pulse, the curve peaks at 117 J. The remaining 5 J left the box before the incoming pulse fully entered the box, mainly in the form of reflected light and partially also in particles. The brown solid curve represents the energy stored in the electromagnetic fields, both the laser pulse and electrostatic fields. The other solid curves represent the energy transferred to the different particle species. A significant fraction of the laser pulse energy is absorbed by the electrons of the foam layer, up to 32 J. Notably, a significant part of this fraction (4.3 J) is carried by very high-energy electrons (above 100 MeV) accelerated via direct laser acceleration. Protons acquire 7.3 J of energy till the fastest population leaves the simulation box. In addition, in total 12.8 J are emitted in the form of photons of energy above 1.022 MeV, of which 12.4 J are emitted by the electrons from the foam, predominantly by nonlinear inverse Compton scattering, and less significantly by betatron radiation emitted during the direct laser acceleration. In these regards, it is worth noticing that there are applications which require simultaneous proton and photon sources^42,43^.

**Fig. 4:**
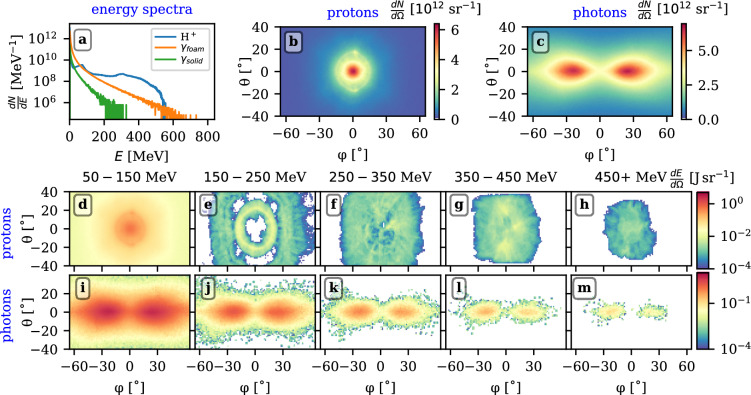
Spectra of accelerated protons and emitted photons. **a**) Energy spectra of protons (blue) and photons emitted by electrons from foam (brown) and solid (green) layers. **b**, **c**) Angular spectra of protons and photons, respectively. **d**-**h**) Angular distribution of energy carried in protons in respective energy bins given in titles. i-m) Similar for photons. The angles are defined as $\varphi = \arctan \frac{p_y}{p_x}$ and $\theta =\arctan \frac{p_z}{\sqrt{p_x^2+p_y^2}}$, respectively. Such a choice assures that for $\varphi =0$, $\theta =0$, the particle propagates along the laser axis. Having $\theta =0$ and $\varphi$ non-zero means emission along the $y-$axis (polarisation direction).

Figure 4 concludes the properties of accelerated protons and also emitted photons at the time of $t=175$ fs, i.e. in the moment when the fastest protons approach the right edge of the box. In particular, the energy spectra of protons (blue) and photons emitted by electrons from the foam (brown) and solid (green) layers are depicted in panel a). The proton and photon peak energies reach up to 557 MeV and approximately 730 MeV, respectively. Their angular distributions are shown in panels b) and c), respectively. The angles $\varphi$ and $\theta$ are defined in the caption. The emission of protons is cylindrically symmetric, peaking along the axis at the value of $6.4\times 10^{12}$ sr^-1^. The full-width half maximum (FWHM) of the divergences considered as the slices along the $\varphi$ and $\theta$ axes are $17.2^{\circ }$ and $17.3^{\circ }$, respectively. A characteristic circular component is apparent at $\sqrt{\varphi ^2+\theta ^2}=17^\circ$ originating from the fact that the hole was born in the solid layer.

On the contrary, photons emit in the characteristic lobes symmetrically positioned along the polarization direction *y*, i.e. $\theta =0$, centered at $\varphi =\pm 23.5^\circ$. The peak emission is $6.5\times 10^{12}$ sr^-1^ and characteristic FWHM of divergences of both lobes are $D_\varphi \approx 52^\circ$ and $D_\theta = 22.8^\circ$. These lobes represent an imprint of the electron bunches accelerated by the direct laser acceleration in the foam. The dominant mechanism leading to the proton emission is the non-linear Compton scattering which occurs when these bunches interact with the laser pulse part reflected by the foil. Of lesser importance, some photons are emitted as synchrotron radiation during the electron acceleration in the NCD layer.

The following panel groups (d-h) and (i-m) of Fig. 4 present angular dependencies of energy emitted by protons and photons, respectively, separated into five energy ranges. In particular, it is worth noticing that the ring pattern clearly visible mainly in panel e), often being observed during experimental campaigns^44–46^, is composed of relatively slow protons, here in the range of [150, 250] MeV. Interestingly, within this energy range, no protons are accelerated along the laser axis, unlike those with lower or higher energies. The lower energy protons are accelerated via the TNSA mechanism, driven by hot electrons originating from the foam layer, which reach the rear of the solid layer already before the laser pulse hits the solid foil. In contrast, the higher energy protons are the result of more complex acceleration mechanisms that arise afterwards.

The respective energy bins of panels (d-h) contain $[15.7, 3.6, 4.0, 2.0, 0.27]\times 10^{10}$ protons carrying a total energy of [2.04,1.14, 1.92, 1.23, 0.20] J, respectively. Indeed, as evident from the energy spectrum of panel a), the decrease in the proton number with the energy is slower than the typical TNSA. In other words, a greater fraction of the accelerated protons is accelerated to higher energies. For example, the conversion efficiency from the laser pulse energy to protons with energy higher than 250 MeV is as high as 2.75%. Similarly, the divergence of the photon lobes decreases with the increase of the energy. For the same energy bins, [$1.6\times 10^{11}$, $1.1\times 10^{10}$, $1.7\times 10^{9}$, $3.2\times 10^{8}$, $6.3\times 10^{7}$] photons carry an energy of [1.9, 0.32, 0.076, 0.021. 0.0050] J. It is necessary to mention that the photons emitted by the Bremsstrahlung are neglected in our simulation. Such an approach is justified as NICS dominates over the Bremsstrahlung already for intensities above $3\times 10^{21}$ W cm^-2^^47,48^, that is much below the regime investigated here.

**Fig. 5:**
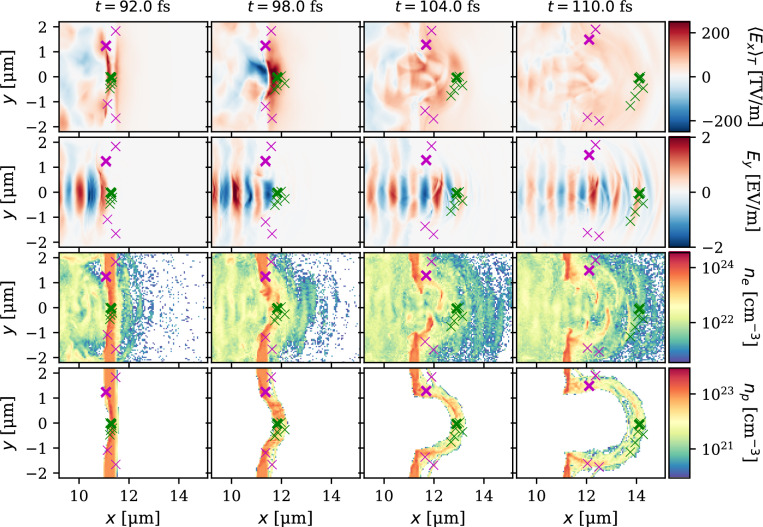
Proton acceleration dynamics. Evolution of the fields $\langle E_x \rangle _T$ (first row), $E_y$ (second row) and densities of electrons (third row) and protons (fourth row). The green and magenta crosses probe protons moving in the *xy* plane, the former move around the laser axis and gain energy higher than 450 MeV, while the latter ones belong to the ring structure visible in Fig. 4e) and reach the final energies of [150, 200] MeV. Although the acceleration process is cylindrically symmetric, only particles within $|z(t)|< 0.5$ $\upmu$m are considered, to allow for a direct comparison with field and density maps. The thicker crosses indicate the protons scrutinized in Fig. 6.

### Investigation of the acceleration dynamics

The dynamics of the acceleration are inspected in Fig. 5 where the longitudinal electric field averaged over the laser pulse period $\langle E_x(t) \rangle _T = \int _{t-T}^t E_x(t')\,d t'$, the instantaneous electric field in the laser polarisation direction $E_y$, and densities of electrons and protons $n_e$ and $n_p$ are captured in four snapshots at the central part of the slice $z=0$. In addition, the positions of the several macroparticles are marked by crosses, the green ones represent the fastest protons being accelerated over 450 MeV, and the magenta ones are those comprising the ring in the proton angular spectra visible in Fig. 4e). The columns represent four selected time instances. The broader picture of the interaction dynamics is shown in a movie in Supplemental Material (Movie 2) where the fields $E_x$, $E_y$, and $B_z$ together with densities of electrons, carbon ions and protons are displayed over a full time of a simulation, and in a whole complete slice of a simulation box at $z=0$.

The first column captures the moment of $t=92$ fs. At that time instance, two phases of the TNSA originating from the electrons from the NCD (started at $t=62$ fs) and solid (started at $t=88$ fs) layers are already in action, accelerating proton and carbon ions from the target’s rear surface. The protons and carbon ions from the front of the solid layer, are accelerated via the hole-boring RPA mechanism (started at $t=89$ fs) by its electron density wavefront. Among those, the marked green macroparticles are located within less than half of a micron distance from the laser axis.

The second column displays the time of $t=98$ fs when the light-sail RPA mechanism dominates the acceleration. At this time instance, the laser pulse begins to propagate through the solid layer, and relativistically induced transparency is achieved. The critical density front of electrons connected to the hole boring RPA mechanism which reached the rear of the solid layer at $t=94$ fs continues to move along with protons, leaving the carbon ions behind. The light sail RPA phase—characterized by the collective forward motion of the entire solid layer—begins around $t=92$ fs, as evidenced by the shift of its rear boundary. This acceleration stage acts on the protons until approximately $t=101$ fs and persists for the carbon ions up to about $t=115$ fs.

In the third panel ($t=104$ fs), the laser pulse begins diverging through the solid layer aperture and expands in all directions, losing its intensity. The dominant acceleration mechanism here and also in the fourth panel ($t=110$ fs) is the hybrid TNSA/RPA regime. It keeps accelerating protons until approximately $t=125$ fs when the field and electron densities around the protons become weak, i.e. about one order of magnitude smaller than at $t=94$ fs, and it cannot accelerate the protons significantly further.

The ring structure protons, marked by the magenta crosses, are located initially out of the high-intensity region, radially about 1-2 $\upmu$m far from the laser axis. Their main final energy gain origins from the target normal sheath field at the rear side, acknowledging that the part of them has been pre-accelerated inside the solid layer by the edge of the shock related with the hole-boring.

**Fig. 6:**
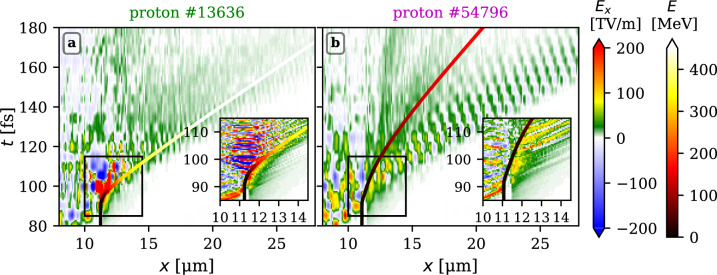
Time history of the electric field $E_x$ along the position *x* of protons marked by thicker crosses in Fig. 5. The trajectories of those protons are depicted by the solid lines line whose colours correspond to protons’ immediate energies. The insets display the same quantity with a better resolution diagnostic within the black rectangles of the main panel.

The acceleration process of the two selected protons (marked by the thicker crosses in Fig. 5) is further scrutinised in Fig. 6. The lines changing colour from black through brown, red, and yellow to white represent the trajectory of the probe protons in the (*x*, *t*) space where the colours correspond to the instantaneous energy. The panels show the profiles of the longitudinal electric field $E_x$ at a given time and along a moving line parallel to the laser axis and always crossing the instantaneous position of the probe proton. The insets show the same quantities, with a better resolution highlighting the region around the centre of the solid layer about the time the laser drills a hole through (indicated by black rectangles in the main axes in each panel). The fast proton (panel a) originates from the front part of the solid layer. It experiences an intense average accelerating field higher than 160 TV/m in the time interval [92, 99] fs, with the peak value exceeding 400 TV/m. Later, it remains in the generally accelerating target normal sheath field modulated by the longitudinal component of the transmitted laser pulse field. Indeed, the acceleration process at this latter stage is shaky, meaning that the rapid acceleration phase is gradually substituted by a phase where the proton is even slightly decelerated, as the laser pulse overtakes the weakly relativistic proton cloud. A lower energy proton shown in panel b) originates from the target front and travels across the solid overdense layer pushed by the edge of the shock, reaching the rear side. Further, it is accelerated by the TNSA field which is significantly weaker compared to panel a), being also modulated by the laser pulse, temporarily even reaching slightly negative values.

**Fig. 7:**
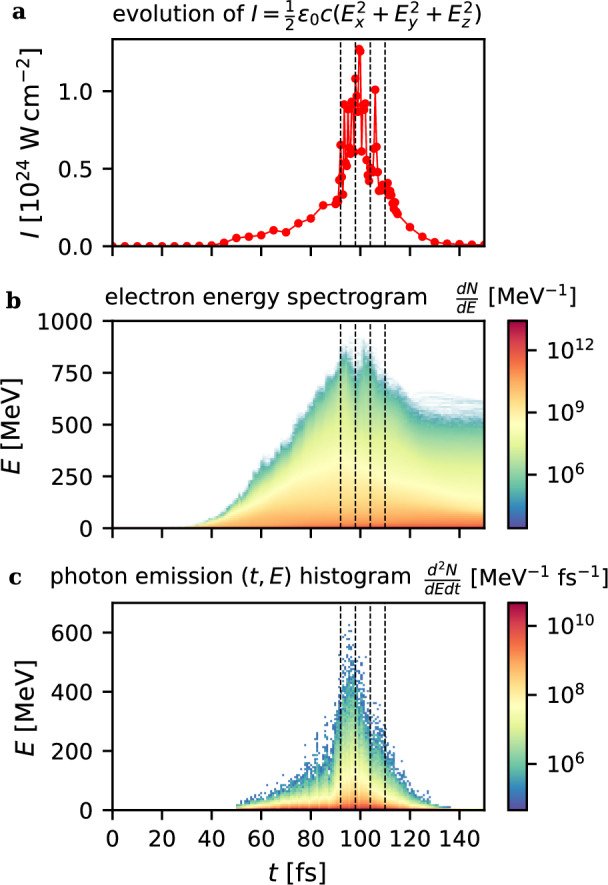
Correlation of field, electron spectra, and photon emission. **a**) Evolution of the value approximating the peak intensity achieved. **b**) Electron energy spectrogram. **c**) (*t*, *E*) histogram of the photon emission. Dashed vertical lines correspond to time instances displayed in Fig. 5.

A complementary illustration of the interaction dynamics is presented in Fig. 7, where the correlation between fields, electrons, and photons is shown. Panel (a) displays the evolution of the peak intensity parameter $I=\tfrac{1}{2}\varepsilon _0 c \textbf{E}^2$. The maximum value of $1.27\times 10^{24}$ W cm^-2^ is reached at $t=99.5$ fs, i.e. 28$\times$ higher than the initial $I_0$, and about 4.2$\times$ higher than the $I_{\text {max}}$ obtained for $n_{e,1}=10n_c$ in Fig. 2c). The observed fourfold increase is a natural consequence of the standing-wave structure formed by interference between the incident and reflected laser pulses. Additional amplification arises from intense electrostatic fields within the plasma.

Panel (b) shows the time evolution of electron energy spectra. The high-energy tail extends up to $\sim$925 MeV at $t=96$ fs, with electrons above 900 MeV sustained over $t\in [95,101]$ fs.

Panel (c) presents the temporal–energy distribution of photon emission. The photon yield correlates closely with both the intensity peak and the appearance of the fastest electrons. Radiation is strongest during $t\in [92,98]$ fs, when the most intense part of the laser pulse interacts with a target that is not yet relativistically transparent. From the standpoint of photon generation, the highest efficiency is expected in the configuration with the greatest laser intensification ($n_{e,1}=25n_c$, $L_1=4.9,\upmu$m; see Fig. 2c), while the solid layer thickness can be considered semi-infinite.

## Conclusion

We investigated laser-driven proton acceleration within the parameter regime achievable with contemporary technology using fully resolved 3D particle-in-cell simulations. Our results demonstrate that protons can be accelerated to energies of up to 550 MeV by interacting a 4.5 PW laser pulse with a structured target composed of a near-critical density (NCD) layer attached to a solid substrate. The thicknesses and densities of both layers are optimized to enhance the laser intensity within the NCD layer and maximize proton acceleration in the solid layer.

The intensified laser pulse drives a shock propagating through the solid, initiating the hole-boring RPA phase. This phase ends before complete reflection, allowing a seamless transition to the highly efficient light sail RPA phase. Additionally, the subsequent hybrid RPA/TNSA phase, which occurs after the accelerated plasma front is penetrated by the laser, is enhanced by hot electrons continuously supplied from the front NCD layer. Overall, up to 2.8% of the laser energy is converted into protons with energies exceeding 250 MeV.

We also identified the origin of a ring structure observed in the proton angular spectrum, attributing it to the compromised TNSA mechanism occurring behind the shot-through target. This phenomenon is often unintentionally observed in experiments using thin targets and insufficient laser contrast. Furthermore, our simulations reveal that approximately 10% of the laser energy is converted into x-rays with a spectrum extending up to 730 MeV via nonlinear inverse Compton scattering.

## Methods

### 3D simulations of laser intensification in the near-critical plasma

A set of three-dimensional particle-in-cell simulations with the code smilei^39^ is conducted by varying the plasma density. The box size is $L_x\times L_y \times L_z$ = 19.2 $\upmu$m$\times$17.6 $\upmu$m$\times$17.6 $\upmu$m. The grid resolution is 25 nm in all dimensions, hence the number of the grid points is 768$\times$704$\times$704. In case of lower densities, $L_x$ is increased. The time step is set to 45.74 as, corresponding to Courant–Friedrichs–Lewy number of 0.95. The simulations always run until after the maximum laser intensity in the NCD layer is reached.

The laser pulse is linearly polarised along the $y-$axis, and has a Gaussian profile in both temporal and spatial domains with characteristic duration at full width-half maximum and minimum waist of $\tau =25$ fs and $w_0 = 2.55$ $\upmu$m, respectively. It delivers the energy $\mathcal {E}_{l,0}=122$ J, leading to a peak power of 4.6 PW and the intensity of $4.5\times 10^{22}$ W cm^-2^. The plasma is modelled by electron and carbon macroparticles, always 4 macroparticles of each species are placed in the corresponding cells.

### 3D simulations of proton acceleration

Here, the box has size of $L_x\times L_y \times L_z$ = 28.8 $\upmu$m$\times$17.6 $\upmu$m$\times$17.6 $\upmu$m, with a grid resolution of 12.5 nm in all dimensions, and hence 2304$\times$1408$\times$1408 grid points. This resolution is fine enough to resolve the skin depth of the solid-density component without the need to artificially reduce its density. The time step is set to 22.87 as, corresponding to Courant–Friedrichs–Lewy number of 0.95. The simulation runs for 7901 steps, corresponding to 180.7 fs. The laser parameters are the same as in the previous section. The target consists of the NCD layer combined with a solid substrate. The lengths and the electron densities of both layers are $L_1=9.6$ $\upmu$m and $n_{e,1}=10n_{c}$, and $L_2=450$ nm and $n_{e,2}=183.6 n_{c}$, respectively. The rear layer density corresponds to a high-density polyethylene (-[CH_2_]-) with a mass density of 0.97 g cm^-3^. The front of the NCD layer is placed at $x=1.4$ $\upmu$m. The layer is modelled by electron and carbon macroparticles, always 4 macroparticles of each species are placed in the corresponding cells. The latter layer is attached directly to the NCD layer. There, electron, hydrogen, and carbon macroparticles are initialised with 8 particles per cell each. In our simulations, both layers are modelled as a fully ionized, homogeneous plasma. Both layers are initialised only at $r=\sqrt{y^2+z^2}< 7.3$ $\upmu$m.

## Supplementary Information

Supplementary Information 1.

Supplementary Information 2.

Supplementary Information 3.

## Data Availability

All data needed to evaluate the conclusions in the paper are present in the paper. Simulation results are available from the corresponding author upon reasonable request.
