# Supplementary material for: Multi-PW laser–driven proton acceleration using a plasma-lens target
Source: Sci Rep. 2025 Dec 6;16:462. doi: 10.1038/s41598-025-29793-7 (PMC12775390; doi:10.1038/s41598-025-29793-7)
Supplement: Supplementary file 3 — Supplementary Information 3. [file 41598_2025_29793_MOESM3_ESM.pdf]

Multi-PW laser–driven proton acceleration using a plasma-lens target

Vojtěch Horný[1, 2, *] and Domenico Doria[1]
[1] *Extreme Light Infrastructure - Nuclear Physics, IFIN-HH, 30 Reactorului Street, 077125 Magurele, Romania*
[2] *Faculty of Nuclear Sciences and Physical Engineering, Czech Technical University in Prague, 115 19 Prague, Czech Republic*
(Dated: October 16, 2025)

PRELIMINARY 3D SIMULATIONS SUPPORTING THE CHOICE OF THE PLASMA LAYER PARAMETERS

For practical purposes, a set of lower-resolution simulations has been launched before proceeding with the one presented in Section III in the main text. The box is of the same size of $L_x \times L_y \times L_z =$ 28.8 µm×17.6 µm×17.6 µm. The grid resolution is 25 nm in all dimensions, hence the number of the grid points is 1150×704×704. The time step is set to correspond to the Courant–Friedrichs–Lewy number of 0.95.

The results of this simulation set are shown in Figure S1, visualizing the evolution of the fastest proton energy found within the simulation box in time. We proceeded as follows. First, the NCD layer density $n_{e,1}$ varied, and its length L_1 is taken as the intensification optima investigated in Section II. The value of the solid density thickness L_2 was calculated from Brantov condition (Eq. 1 in the main paper, and Ref. [1]). These results are presented in panel a), showing that there exists an optimum for the proton acceleration, namely, the NCD layer density and thickness of $n_{e,1} = 10n_c$, $L_1 = 9.6$ µm, and the corresponding solid layer thickness of $L_2 = 825$ nm (olive line). It is worth noticing that this optimum does not correspond to the best laser pulse intensification case which is achieved by $n_e = 25n_c$ (cf. Fig 2a in the main paper). Indeed, laser pulse energy losses via absorption and reflection in such a denser plasma deteriorate the proton acceleration significantly, outbalancing the potentially anticipated enhancing effect of the higher pulse intensity. A blue line representing the standard single-layer case is also shown for a direct comparison. It indicates rather strong acceleration up to 475 MeV. Nevertheless, let us point out that such a thin layer, in practice, could barely sustain the impact of the prepulses and intensity rising edge preceding the main pulse, which would transform that foil into the thicker plasma cloud of an on average lower density, compromising the advanced radiation pressure acceleration processes.

In panel b), the same dependence is shown for the cases with the optimum foam layer parameters $n_{e,1} = 10n_c$ and $L_1 = 9.6$ µm, however, the thickness of the solid layer L_2 is varied. The optimum acceleration is achieved for $L_2 = 0.45$ µm (pink). The higher resolution simulation of this case is presented in the main paper. Here, the peak energy reaches up to 568 MeV, i.e. by a factor of 1.02 more than in the fully resolved simulation. Again, for comparison, the olive curve representing the thickness

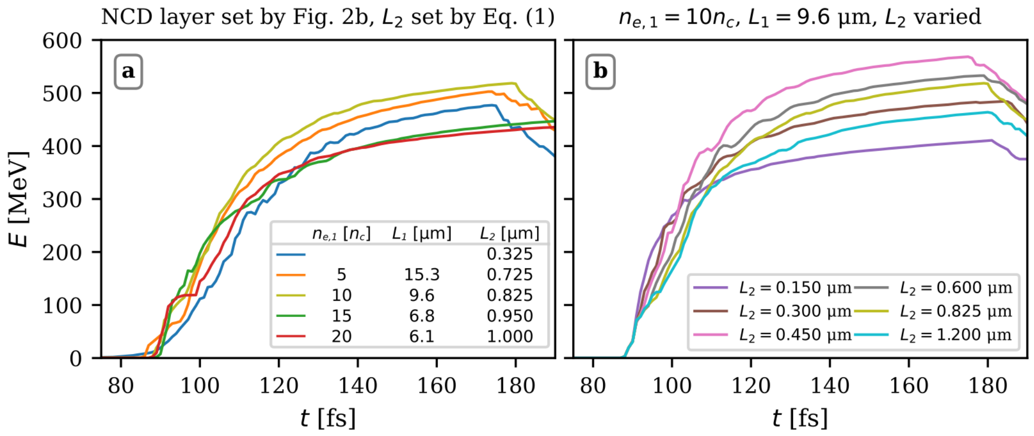

Fig. S1. Evolution of the energy of the fastest proton in the simulation box with time for different configurations of front layer density and both layer thicknesses. a) Values of $n_{e,1}$ and L_1 varied according to optima found in Fig. 2b in Section II, L_2 chosen from Brantov condition. b) $n_{e,1} = 10n_c$, $L_2 = 9.6$ µm, L_2 varied.

$L_2 = 0.825$ μm given by the Brantov condition is plotted as well.

The explanation for this difference can be understood by analyzing the curves in panel b. As described in Section III of the main paper, the fastest protons are accelerated through a sequence of acceleration phases: starting with hole-boring radiation pressure acceleration (HB-RPA), followed by light sail RPA (LS-RPA), and concluding with a hybrid TNSA/RPA phase. In general, HB-RPA is less efficient than LS-RPA.

For cases with $L_2 < 450$ nm, the less efficient HB-RPA phase is shorter, allowing the transition to the more efficient LS-RPA phase to occur earlier and at a higher remaining pulse intensity. Consequently, the purple and brown curves lie above the pink curve in the time range of approximately [89, 101] fs. For the pink curve, the LS-RPA phase ends around the latter time, whereas for the thinner and thicker targets, this phase ends a few femtoseconds earlier and later, respectively.

Once the LS-RPA phase ends, a significant portion of the laser light penetrates through the plasma front in cases with $L_2 < 450$ nm, leading to less efficient acceleration during the subsequent hybrid TNSA/RPA phase. Conversely, for $L_2 > 450$ nm, the initial HB-RPA phase is longer and less efficient, delaying the onset of LS-RPA, which then occurs with a lower light intensity. As a result, even though the final hybrid TNSA/RPA phase contributes to the energy gain, it is insufficient to compensate for the earlier energy deficit.

Although the greatest energy gain is achieved during the initial RPA phases, the acceleration in the later hybrid TNSA/RPA phase remains significant. Unlike in the standard single-layer configuration discussed in Ref. [1], the double-layer configuration enhances this stage's efficiency because hot electrons necessary for the TNSA mechanism are continuously supplied by the front NCD layer. Therefore, the optimum thickness solid density layer thickness in the double layer configuration is lower than the optimum foil thickness in a single layer target case.

Nevertheless, a comparison of all the curves from both panels of Figure S1 reveals that the final energy of the most energetic protons remains relatively consistent across a wide range of double-layer target configurations. This robustness is a desirable feature for practical applications.

NUMERICAL COST OF THE PERFORMED SIMULATION

This simulation described in Section III has been conducted at 160 nodes of the Karolina supercomputer located in the IT4Innovations facility in Ostrava, Czechia. Each computation node consists of 2× AMD EPYC 7H12, 64-cores, 2.6 GHz processors per node. Hence, in total, the simulation ran on 20,480 CPUs. Only such a large number of nodes satisfied the memory demands which manifested mainly when writing 3D diagnostic outputs used for visualization purposes. This simulation ran for 10 hours and 18 minutes, total CPU time demands were then 211,000 CPU hours, corresponding to 500 kWh of electric energy.

* vojtech.horny@eli-np.ro

[1] A. Brantov, E. Govras, V. Y. Bychenkov, and W. Rozmus, Ion energy scaling under optimum conditions of laser plasma acceleration from solid density targets, Physical Review Special Topics-Accelerators and Beams **18**, 021301 (2015).
